# Novel Indices of Glucose Homeostasis Derived from Principal Component Analysis: Application for Metabolic Assessment in Pregnancy

**DOI:** 10.1155/2020/4950584

**Published:** 2020-03-30

**Authors:** Tina Stopp, Michael Feichtinger, Ingo Rosicky, Gülen Yerlikaya-Schatten, Johannes Ott, Hans Christian Egarter, Christian Schatten, Wolfgang Eppel, Peter Husslein, Martina Mittlböck, Andrea Tura, Christian S. Göbl

**Affiliations:** ^1^Department of Gynecology and Obstetrics, Division of Feto-Maternal Medicine, Medical University of Vienna, Vienna, Austria; ^2^Wunschbaby Institut Feichtinger, Vienna, Austria; ^3^Department of Obstetrics and Gynecology, Division of Gynecologic Endocrinology and Reproductive Medicine, Medical University of Vienna, Vienna, Austria; ^4^Center of Medical Statistics, Informatics, and Intelligent Systems, Section for Clinical Biometrics, Medical University of Vienna, Vienna, Austria; ^5^Metabolic Unit, CNR Institute of Neuroscience, Padova, Italy

## Abstract

**Aims:**

This study is aimed at assessing the association of previously developed indices of glucose homeostasis derived from principal component analysis (PCA) with parameters of insulin action, secretion, and beta cell function during pregnancy.

**Methods:**

In this prospective longitudinal study, an oral glucose tolerance test was performed in sixty-seven pregnant women at two prepartum (12+0 to 22+6 and 24+0 to 28+6) and one postpartum (2 to 11 months) visits. Three principal component scores (PCS) were calculated based on measurements of glucose, insulin, C-peptide, age, and BMI to assess their association with fasting and dynamic indices of insulin action, secretion, and *β*-cell function.

**Results:**

PCS1 was positively associated with fasting and dynamic parameters of insulin sensitivity (Matsuda index: *r* = 0.93, *p* < 0.001), whereas a strong negative association was observed for early, late, and total insulin response. PCS2 was associated with higher mean glucose but negatively related to parameters of insulin secretion. PCS3 was significantly associated with fasting indices of insulin sensitivity. PCS1 to 3 assessed at early pregnancy were also associated with development of GDM, whereby random forest analysis revealed the highest variable importance for PCS1. PCS1 to 3 were significantly related to the oral disposition index explaining 49.0% of its variance.

**Conclusions:**

PCS1 to 3 behaved similarly as compared to previous observations in nonpregnant women and were furthermore associated with the development of GDM. These findings support our hypothesis that PCS1 to 3 could be used as novel indices of glucose disposal during pregnancy.

## 1. Introduction

Since the Hyperglycemia and Adverse Pregnancy Outcome (HAPO) study has illustrated a continuous relationship between glucose levels measured during the oral glucose tolerance test (OGTT) and adverse pregnancy outcomes [1], the OGTT is gaining importance for the diagnosis of gestational diabetes mellitus (GDM) [2]. However, the clinical information obtained from this test can be notably increased by the dynamic assessment of insulin and C-peptide in addition to plasma glucose concentrations. For this purpose, several indices have been developed to describe the amount of insulin action and secretion, aimed at providing more detailed information on glucose disposal [3]. Recently, we introduced principal component analysis (an unsupervised machine learning algorithm) as a novel approach to explain the dominant patterns of dynamic OGTT measurements [4]. Thereby, three principal components were identified which explained 71.5% of the total variance of glucose, insulin, and C-peptide concentrations measured during a 2 h OGTT at three to six months after delivery. The subsequently calculated principal component scores (PCS1 to PCS3) were closely related to parameters of glucose disposal. In particular, PCS1 and PCS3 were associated with dynamic and fasting indices of insulin sensitivity, respectively, whereas PCS2 was shown to be associated with *β*-cell failure. All three principal component scores were predictive for the later development of type 2 diabetes in women with a history of GDM, indicating that those scores might be appropriate surrogates for glucose metabolism and useful for risk stratification in the postpartum period.

This study is aimed at evaluating the performance of these novel indices during the gestational period by using an independent study population. For this purpose, principal component scores were calculated by using the eigenvectors of our previous study to quantify their association with traditional parameters of glucose metabolism to gain further insight into pathophysiologic processes as a primary objective. As a secondary objective and to give an outlook on a potential future clinical application, their association with development of GDM was examined.

## 2. Participants and Methods

### 2.1. Study Design and Participants

For this prospective longitudinal study, sixty-seven pregnant women (Caucasian: *n* = 62, Asian: *n* = 4, and South American: *n* = 1) were consecutively recruited between June 2015 and September 2017. A broad characterization with anthropometric parameters as well as detailed metabolic assessments was performed between 12+0 and 22+6 weeks of gestation (V1; mean: 18.1 ± 2.6 weeks) and repeated between 24+0 and 28+6 weeks of gestation (V2; mean: 26.4 ± 1.4 weeks) as well as 2 to 11 months after delivery (V3; mean: 4.3 ± 3.1 month). Twenty-three women showed a positive family history of type 2 diabetes (first or second degree). At each visit, an OGTT was performed including measurements of glucose, insulin, and C-peptide at fasting and every 30 minutes for 120 minutes following a 75 g oral glucose load. Six women were diagnosed with GDM according to the International Association of the Diabetes and Pregnancy Study Groups (IADPSG) recommendations: *fasting* *plasma* *glucose* ≥ 5.1 *mmol*/*l* or 1‐*hour* *plasma* *glucose* ≥ 10.0 *mmol*/*l* or 2‐*hour* *plasma* *glucose* ≥ 8.5 mmol/l following a 75 g oral glucose load at 24 to 28 weeks of gestation [2]. Four patients were diagnosed at V2. Two patients met the IADPSG thresholds already at V1 and were classified as GDM due to elevated self-monitored blood glucose levels during follow-up. Patients with preexisting diabetes were excluded. The study was approved by the Ethics Committee of the Medical University of Vienna and performed in accordance with the Declaration of Helsinki. All participants gave written informed consent.

### 2.2. Calculation of Principal Component Scores

PCS were calculated as the product of two input matrices: *A* × *V* = *S*. *A* is a matrix of centred and standardized data of the actual measurements of glucose, insulin (after log transformation), and C-peptide (after square root transformation) as well as age and BMI (after log transformation) and *V* is a matrix containing the loadings of each variable on the first three principal components, i.e., the matrix containing the eigenvectors derived from our previous publication [4]. To obtain meaningful indices, the derived PCS were subsequently rescaled by (*S* + 100)/17, where *S* is the output matrix on the original scale. The loading matrix *V* is provided as supplemental material ([Supplementary-material supplementary-material-1]). The original study included 151 women (110 with history of GDM and 41 with normal glucose tolerance during gestation).

### 2.3. Calculation of Parameters of Glucose Homeostasis

In addition, dynamic indices of insulin action were assessed from OGTT data containing the oral glucose insulin sensitivity index (OGIS), the Matsuda index (ISI-Matsuda), and Stumvoll's parameters of metabolic clearance rate (MCR) and insulin sensitivity (ISI) [5–7]. The homeostasis model assessment of insulin resistance (HOMA-IR) as well as the quantitative insulin sensitivity check index (QUICKI) was used to examine insulin sensitivity at fasting [8, 9]. Insulinogenic indices were used to describe early (Sec-early: *Δ*I30-0/*Δ*G30-0), late (Sec-late: AUC_Insulin_/AUC_Glucose 60-120_), and overall insulin response to glucose (Sec-total: AUC_Insulin_/AUC_Glucose 0-120_) during the OGTT [10] in addition to approximations of first- (PH1) and second-phase insulin response (PH2) [6]. The oral disposition index (ISSI-2) was calculated as the product of ISI-Matsuda and AUC_Insulin_/AUC_Glucose 0-120_ to reflect the extent to which *β*-cells can adapt to impaired insulin action. Specific parameters describing different aspects of *β*-cell function, such as glucose sensitivity (G-sens, representing the mean slope of dose response of insulin release on glucose levels at any time point during the OGTT), rate sensitivity (rate-sens, representing early insulin release on the rate of change of glucose concentrations), and total insulin release from C-peptide (TIS), were additionally assessed [11].

### 2.4. Statistical Analysis

Categorical variables were summarized by counts and percentages and compared by Pearson's Chi-squared test. Continuous variables were summarized by means and standard deviations (SD). If single measurements during the OGTT were missing (5 cases), multivariate imputations by chained equations were used to estimate the missing values by the average of *m* = 50 complete data sets. Appropriate data transformations such as square root or natural logarithm were applied to normalize the data if skewed distribution was detected by descriptive analysis. Comparison of PCS between NGT and GDM women at the first visit was performed by Welch's *t*-test. Linear regression as well as Spearman's rank correlation was used to assess the association between PCS and parameters of interest at different visits. To account for correlated residuals, linear mixed effects models were used. Univariable binary logistic regression was used to assess the association between PCS and the development of GDM. Thereby, 95% confidence intervals (95% CI) were estimated by using the likelihood ratio statistic. Moreover, random decision forests with *n*tree = 10^6^ were created by the conditional inference framework (cforest) to derive measures of variable importance as the average difference in predictive accuracy before and after permutation of a predictor variable over all (i.e., one million) trees [12, 13].

Statistical analysis was performed by R (V 3.5.1) and contributing packages. *p* values ≤ 0.05 (two-sided) were considered statistically significant.

## 3. Results

### 3.1. Association of Principal Component Scores with Parameters of Glucose Metabolism

**Figure 1:**
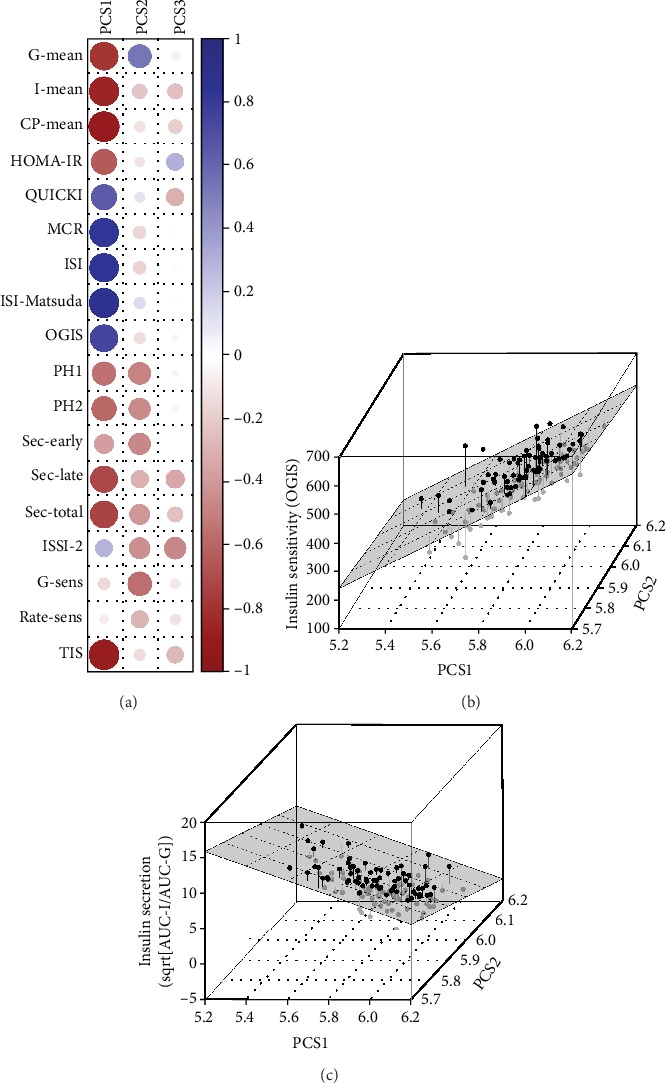
Correlation map representing the amount of association of glucometabolic parameters with principal component scores (PCS1, PCS2, and PCS3). Size of the circle and color intensity are proportional to the amount of correlation (dark color representing higher Spearman's correlation coefficient; positive = blue, negative = red) (a). Three-dimensional visualization of the association of the first two principal components (PCS1 and PCS2) with insulin sensitivity (OGIS) (b) and insulin secretion (sqrt[AUC-I/AUC-G) (c).

A summary of the data used for the calculations of the principal component scores including age, BMI, and OGTT measurements is provided in Table 1. PCS1 was positively associated with fasting and dynamic indices of insulin sensitivity, whereas a strong negative association was observed for early to late and total insulin response to glucose (Figures 1(a)–1(c)). In addition, we observed a close negative association with mean glucose, insulin, and C-peptide concentrations (Figure 1(a)). In contrast, PCS2 was associated with higher mean glucose but (comparable to PCS1) inversely related to parameters of insulin response as visualized in Figures 1(a) and 1(c). PCS3 was inversely associated with fasting indices of insulin sensitivity (Figure 1(a)). As a consequence, increased PCS1 was associated with improved *β*-cell function (*b* = 0.87, 95% CI 0.49 to 1.35), whereas an increase in PCS2 (*b* = −1.38, 95% CI -2.50 to -0.47) and PCS3 (*b* = −2.74, 95% CI -3.73 to -1.52) reflected a decrease of the oral disposition index (i.e., ISSI-2). This model explained 49.0% of the ISSI-2 variance at V1, and comparable results were observed when all visits were analyzed together. These conclusions remained constantly valid in a sensitivity analysis after excluding five patients with non-Caucasian origin.

### 3.2. Differences between the Visits and Longitudinal Evaluation

Correlation analyses for PCS1-PCS3 and parameters of glucose metabolism separated for each visit are provided in the supplemental material (Tables [Supplementary-material supplementary-material-1]). The correlation was comparable between the visits, although it is worth mentioning that the association between PCS3 and fasting indices of insulin action (HOMA-IR and QUICKI) was stronger after delivery. As shown in Table 1, PCS1 and PCS3 levels changed during the study period (PCS1 decreased from V1 to V2 and increased after delivery, and PCS3 increased after delivery), whereas no pregnancy-related changes were observed for PCS2.

### 3.3. Association of Principal Component Scores with GDM Development

Six incident cases of GDM were identified. Demographic characteristics of GDM patients compared to NGT patients are shown in Table 2. Univariable binary logistic regression indicated that principal component scores assessed at V1 (PCS1: OR 0.82, 95% CI 0.68 to 0.91, *p* < 0.001; PCS2: OR 1.18, 95% CI 1.05 to 1.37, *p* = 0.004; PCS3: OR 1.14, 95% CI 1.00 to 1.32, *p* = 0.046; all ORs refer to an increase of 0.01 score units) were significantly associated with the development of GDM. A visualization of the association between PCS 1-3 and GDM status is provided in the supplemental material ([Supplementary-material supplementary-material-1]). Random forest analysis revealed higher variable importance for PCS1 as compared to traditional risk factors (age and family history of type 2 diabetes) and other parameters of glucose metabolism (fasting glucose, mean glucose during the OGTT, OGIS, and TIS; Table 3). Lower variable importance metrics were reported for PCS2 and PCS3. However, the limited number of GDM cases should be considered when interpreting these results.

## 4. Discussion

This study is aimed at assessing the performance of PCA-derived novel indices of glucose metabolism during pregnancy, which were previously developed in postpartum women with history of GDM, in terms of the extent of their correlation with traditional fasting and dynamic parameters of insulin resistance and secretion obtained from OGTT data. Thereby, three PCS were calculated by using the eigenvectors as described in our previous publication [4]. The results showed that all three indices PCS1 to 3 behaved similarly as compared to our previous study in postpartum women [4]: PCS1 showed close associations with various indices of insulin sensitivity. Moreover, it was inversely related to parameters of insulin secretion suggesting that it also embodies the adaptation to impaired insulin action, which physiologically occurs in normal glucose-tolerant individuals. PCS2 was also inversely related to parameters of insulin secretion, but in contrast to PCS1, this association was independent of insulin action. Therefore, it is likely that this component rather reflects *β*-cell dysfunction. The correlation between PCS3 and parameters of glucose metabolism was lower as compared to PCS1 and PCS2; however, its positive association with fasting insulin resistance as well as its inverse association with the oral disposition index remained significant over all visits. Of note, women who developed GDM showed altered scores of PCS1 and PCS2 already at early gestation. These findings support our hypothesis that PCS1 to 3 could be used as indices of glucose disposal and GDM risk prediction. Thereby, several advantages of PCS over more traditional indices of glucose metabolism need to be mentioned: PCS are uncorrelated by concept, which is an important aspect for clinical prediction models [14]. In addition, the simultaneous estimation of insulin sensitivity and *β*-cell function by one simple mathematical operation (i.e., product of two matrices) is a further benefit of our method.

Recently, Wagner et al. found that traditionally used indices of insulin action like ISI-Matsuda and OGIS failed to capture the difference in insulin resistance between pregnant and nonpregnant women and proposed an alternative index based on BMI, insulin, and nonesterified fatty acids [15]. While this concept is of interest, the assessment of nonesterified fatty acids may be subject to additional costs and efforts in routine clinical settings as compared to the more routinely assessed fasting and dynamic glucose, insulin, and C-peptide measurements during OGTT. In our longitudinal study, PCS1 (which mainly reflects insulin sensitivity) decreased during gestation and increased after delivery, suggesting that this index, based on routinely measured parameters, adequately reflects the expected changes in insulin action during this period. In contrast, ISI-Matsuda and OGIS did not significantly change from V1 to V2. One possible advantage of PCS1 is that it incorporates the information of serial measurements of C-peptide concentrations in addition to glucose and insulin levels, which are traditionally used in most empirical (e.g., ISI-Matsuda) and model-based indices of insulin action (e.g., OGIS). Of note, C-peptide concentrations have more constant peripheral clearance with higher and more stable blood concentrations [16] and in contrast to insulin levels showed an expected increase during gestation (Table 1). PCS3 (which is much strongly related to fasting levels than PCS1) increased after delivery, explainable by physiologically lower fasting glucose concentrations during pregnancy. Therefore, differences in the absolute values of this score should be interpreted with caution especially when pregnant and nonpregnant individuals are compared. However, it has to be mentioned that PCS3 was also significantly associated with parameters of glucose disposal including ISSI-2 at any time point in our study, suggesting that it embodies relevant information on glucose metabolism independently of both other scores as well.

We conclude that our novel indices could be useful for the assessment of glucose disposal and GDM risk classification during gestation. In line with our previous observations in women after delivery, we identified PCS1 and PCS3 as potential predictors for insulin action, whereby PCS3 rather reflects insulin resistance at fasting condition (i.e., the amount of hepatic insulin resistance). PCS2 mainly mirrors *β*-cell dysfunction. Moreover, we found that our indices assessed at early gestation were closely related to the development of GDM. PCS1 in particular revealed a higher variable importance as compared to other parameters. While this study represents the first attempt to evaluate principal component scores in the gestational period as possible indices for impaired glucose metabolism, the restricted number of study participants who developed GDM as well as the lack of generalizability of our results to other ethnicities must be noted as a limitation of this work. Thus, we emphasize to further address the possible advantage of our proposed indices in larger populations with different ethnic backgrounds.

## Figures and Tables

**Table 1:** Data of the study sample used for calculating the principal component scores at 12+0 to 22+6 (V1), 24+0 to 28+6 weeks of gestation (V2), and 2 to 11 months after delivery (V3).

	*n*	V1	*n*	V2	*n*	V3	*p* value^§^
Age (years)	67	29.8 ± 5.1	62	30.1 ± 4.8^#^	25	31.9 ± 5.4^#^	<0.001
BMI (kg/m^2^)^∗^	67	3.24 ± 0.18	57	3.31 ± 0.16^#^	23	3.22 ± 0.14	<0.001
BMI (kg/m^2^)^†^	67	26.0 ± 5.0	57	27.7 ± 4.6^#^	23	25.2 ± 3.4	<0.001
G0 (mg/dl)	67	76.8 ± 7.5	61	77.8 ± 9.3	23	83.0 ± 9.3^#^	0.003
G30 (mg/dl)	67	125.1 ± 25.5	59	129.7 ± 25.4	23	126.4 ± 21.8	0.181
G60 (mg/dl)	67	118.5 ± 33.4	59	131.9 ± 31.8^#^	23	110.4 ± 29.6	<0.001
G90 (mg/dl)	67	102.8 ± 29.6	59	114.5 ± 29.8^#^	23	91.9 ± 20.8	<0.001
G120 (mg/dl)	67	97.1 ± 24.5	59	102.7 ± 25.6	23	94 ± 18.0	0.055
I0 (*μ*U/ml)^∗^	67	1.84 ± 0.83	59	1.86 ± 1.04	23	1.00 ± 1.41^#^	0.001
I30 (*μ*U/ml)^∗^	67	3.62 ± 1.34	57	3.46 ± 1.57	23	3.00 ± 1.30	0.239
I60 (*μ*U/ml)^∗^	67	3.63 ± 1.24	57	3.63 ± 1.32	23	3.14 ± 1.09	0.157
I90 (*μ*U/ml)^∗^	67	3.29 ± 1.38	57	3.60 ± 1.28	23	3.04 ± 1.06	0.104
I120 (*μ*U/ml)^∗^	67	3.28 ± 1.35	57	3.50 ± 1.14	23	2.52 ± 1.04^#^	<0.001
CP0 (ng/ml)^∗∗^	67	1.30 ± 0.25	59	1.43 ± 0.27^#^	23	1.30 ± 0.27	<0.001
CP30 (ng/ml)^∗∗^	67	2.48 ± 0.43	57	2.60 ± 0.50^#^	23	2.34 ± 0.34	0.015
CP60 (ng/ml)^∗∗^	67	2.80 ± 0.53	57	3.10 ± 0.55^#^	23	2.62 ± 0.34	<0.001
CP90 (ng/ml)^∗∗^	67	2.71 ± 0.59	57	3.06 ± 0.63^#^	23	2.59 ± 0.38	<0.001
CP120 (ng/ml)^∗∗^	67	2.61 ± 0.61	57	2.94 ± 0.62^#^	23	2.47 ± 0.44	<0.001
QUICKI (dimensionless)	67	0.17 ± 0.03	59	0.17 ± 0.05	23	0.20 ± 0.07^#^	0.007
ISI-Matsuda (dimensionless)^∗^	67	2.04 ± 0.81	57	1.94 ± 0.85	23	2.70 ± 0.88^#^	<0.001
OGIS (ml/min m^−2^)	67	505 ± 85.1	57	481 ± 86.1	23	486 ± 60.9	0.086
TIS (nmol/m)^∗^	67	3.70 ± 0.37	57	3.92 ± 0.35^#^	23	3.60 ± 0.29	<0.001
PCS1	67	5.90 ± 0.17	57	5.83 ± 0.15^#^	23	5.96 ± 0.11^#^	<0.001
PCS2	67	5.87 ± 0.07	57	5.88 ± 0.09	23	5.91 ± 0.06	0.170
PCS3	67	5.88 ± 0.07	57	5.87 ± 0.06	23	5.91 ± 0.06^#^	0.032

Data are expressed as means ± standard deviations. BMI: body mass index. Values are given for glucose (G), insulin (I), and C-peptide (CP) for fasting as well as 30, 60, 90, and 120 minutes after oral glucose load. QUICKI: quantitative insulin sensitivity check index; ISI-Matsuda: Matsuda index; OGIS: oral glucose insulin sensitivity index; TIS: total insulin secretion from C-peptide; PCS: principal component scores 1–3. ^∗^Log transformation (natural logarithm). ^†^BMI (original units). ^∗∗^Square root transformation. ^#^*p* < 0.05 vs. V1. ^§^Test for global hypothesis: V1 vs. V2 vs. V3.

**Table 2:** Demographic characteristics of NGT subjects compared to GDM subjects.

	*n*	NGT	*n*	GDM	*p* values
Age at enrollment (years)	61	29.08 ± 4.68	6	36.67 ± 4.63	<0.001
Primiparity (total/in % of *n*)	61	41/67.2	6	4/66.6	0.978
Prepregnancy BMI (kg/m^2^)^∗^	61	3.14 ± 0.15	6	3.50 ± 0.28	<0.001
History of GDM (total/in % of *n*)	61	3/4.9	6	2/33.3	0.06
History of GDM (total/in % of *n*) in multiparous women	20	3/15.0	2	2/100.0	0.043
Family history with T2 diabetes (total/in % of *n*)	60^#^	20/33.3	6	3/50	0.413
G0 at V1 (mg/dl)	61	75.4 ± 5.6	6	91.2 ± 9.7	<0.001
G60 at V1 (mg/dl)	61	112.8 ± 29.0	6	176.3 ± 16.5	<0.001
G120 at V1 (mg/dl)	61	93.6 ± 22.2	6	134.4 ± 18.9	<0.001

Data are expressed as means ± standard deviations or counts and percentages of BMI: body mass index; NGT: normal glucose tolerance; GDM: gestational diabetes mellitus; T2 diabetes: type 2 diabetes mellitus. G0, G60, and G120 are glucose values for fasting and 60 and 120 minutes after oral glucose load at V1 (first visit, 12 to 22 weeks of gestation). ^∗^Natural log transformation. ^**#**^One patient was adopted, family history not available.

**Table 3:** Analysis of different explanatory variables for GDM at the first visit (12+0 to 22+6 weeks of gestation).

	OR	95% CI	*p* value	VIMP
PCS1	0.82	0.68-9.10	<0.001	1.54 × 10^−2^
PCS2	1.18	1.05-1.37	0.004	6.67 × 10^−7^
PCS3	1.14	1.00-1.32	0.046	−7.50 × 10^−7^
Age (years)	1.47	1.17-2.06	<0.001	−1.13 × 10^−5^
BMI (kg/m^2^)	1.38	1.16-1.74	<0.001	3.40 × 10^−3^
Family history with diabetes	2.00	0.34-11.7	0.424	0.00
G0 (mg/dl)	1.35	1.15-1.71	<0.001	3.06 × 10^−3^
G-mean (mg/dl)	1.18	1.08-1.41	<0.001	8.17 × 10^−3^
OGIS (ml min^−1^ m^−2^)	0.98	0.96-0.91	<0.001	1.67 × 10^−6^
TIS (nmol m^−2^)	1.09	1.03-1.16	0.002	−3.58 × 10^−5^

Odds ratios (OR), 95% confidence intervals (95% CI), and variable importance measures from random forest analysis (VIMP) are provided for the following: PCS: principal component scores 1–3; BMI: body mass index; G0: fasting plasma glucose levels; G-mean: mean plasma glucose levels during the OGTT; OGIS: oral glucose insulin sensitivity index; TIS: total insulin secretion from C-peptide.

## Data Availability

Data are available from the corresponding author for researchers who meet the criteria for access to confidential data. Please contact Christian Göbl christian.goebl@meduniwien.ac.at.

## Additional Points

*Novelty Statement*. The present study is aimed at assessing the performance of previously developed indices of glucose homeostasis in pregnant women. Our results show that the novel indices behave similarly as compared to our observations in women after delivery. Women who developed gestational diabetes showed altered indices already at early gestation. Our findings support our hypothesis that our novel indices, based on OGTT-derived measurements of glucose, insulin, and C-peptide as well as age and BMI, could be used for the assessment of glucose homeostasis and GDM risk prediction during pregnancy.

## Supplementary Materials

Supplementary Materials Figure S1: box-whisker plots, representing comparisons of principal component scores between women who have normal glucose tolerance (NGT) and those who developed gestational diabetes mellitus (GDM): PCS1 (A); PCS2 (B); PCS3 (C). Table S1: loadings of each variable on the first three principal components (i.e., how strong original variables are related to each principal component), derived from our previous publication [1]. Table S2: correlation of the principal component scores (PCS) with glucometabolic parameters at 12+0 to 22+6 (V1) clearance rate; ISI: insulin sensitivity index; ISI-Matsuda: Matsuda index; OGIS: oral glucose insulin sensitivity index; PH1: first-phase insulin secretion; PH2: second-phase insulin secretion; Sec-early: early insulin response to glucose; Sec-late: late insulin response to glucose; Sec-tot: total insulin response to glucose; ISSI-2: disposition index; G-sens: *β*-cell glucose sensitivity; rate-sens: rate sensitivity; TIS: total insulin secretion from C-peptide. Table S3: correlation of the principal component scores (PCS) with glucometabolic parameters at 24+0 to 28+6 weeks of gestation (V2) clearance rate; ISI: insulin sensitivity index; ISI-Matsuda: Matsuda index; OGIS: oral glucose insulin sensitivity index; PH1: first-phase insulin secretion; PH2: second-phase insulin secretion; Sec-early: early insulin response to glucose; Sec-late: late insulin response to glucose; Sec-tot: total insulin response to glucose; ISSI-2: disposition index; G-sens: *β*-cell glucose sensitivity; rate-sens: rate sensitivity; TIS: total insulin secretion from C-peptide. Table S4: correlation of the principal component scores (PCS) with glucometabolic parameters 2 to 11 months after delivery (V3) clearance rate; ISI: insulin sensitivity index; ISI-Matsuda: Matsuda index; OGIS: oral glucose insulin sensitivity index; PH1: first-phase insulin secretion; PH2: second-phase insulin secretion; Sec-early: early insulin response to glucose; Sec-late: late insulin response to glucose; Sec-tot: total insulin response to glucose; ISSI-2: disposition index; G-sens: *β*-cell glucose sensitivity; rate-sens: rate sensitivity; TIS: total insulin secretion from C-peptide. Table S5: association with development of GDM and variable importance index provided by random forest analysis.

## References

[1] HAPO Study Cooperative Research Group (2008). Hyperglycemia and adverse pregnancy outcomes. *The New England Journal of Medicine*.

[2] International Association of Diabetes and Pregnancy Study Groups Consensus Panel (2010). International Association of Diabetes and Pregnancy Study Groups recommendations on the diagnosis and classification of hyperglycemia in pregnancy. *Diabetes Care*.

[3] Muniyappa R., Lee S., Chen H., Quon M. J. (2008). Current approaches for assessing insulin sensitivity and resistance in vivo: advantages, limitations, and appropriate usage. *American Journal of Physiology-Endocrinology and Metabolism*.

[4] Göbl C. S., Bozkurt L., Mittlböck M. (2015). To explain the variation of OGTT dynamics by biological mechanisms: a novel approach based on principal components analysis in women with history of GDM. *American Journal of Physiology-Regulatory, Integrative and Comparative Physiology*.

[5] Mari A., Pacini G., Murphy E., Ludvik B., Nolan J. J. (2001). A model-based method for assessing insulin sensitivity from the oral glucose tolerance test. *Diabetes Care*.

[6] Stumvoll M., Mitrakou A., Pimenta W. (2000). Use of the oral glucose tolerance test to assess insulin release and insulin sensitivity. *Diabetes Care*.

[7] Matsuda M., DeFronzo R. A. (1999). Insulin sensitivity indices obtained from oral glucose tolerance testing: comparison with the euglycemic insulin clamp. *Diabetes Care*.

[8] Matthews D. R., Hosker J. P., Rudenski A. S., Naylor B. A., Treacher D. F., Turner R. C. (1985). Homeostasis model assessment: insulin resistance and beta-cell function from fasting plasma glucose and insulin concentrations in man. *Diabetologia*.

[9] Katz A., Nambi S. S., Mather K. (2000). Quantitative insulin sensitivity check index: a simple, accurate method for assessing insulin sensitivity in humans. *The Journal of Clinical Endocrinology and Metabolism*.

[10] Tura A., Kautzky-Willer A., Pacini G. (2006). Insulinogenic indices from insulin and C-peptide: comparison of beta-cell function from OGTT and IVGTT. *Diabetes Research and Clinical Practice*.

[11] Mari A., Tura A., Gastaldelli A., Ferrannini E. (2002). Assessing insulin secretion by modeling in multiple-meal tests: role of potentiation. *Diabetes*.

[12] Hothorn T., Hornik K., Zeileis A. (2006). Unbiased recursive partitioning: a conditional inference framework. *Journal of Computational and Graphical Statistics*.

[13] Strobl C., Boulesteix A.-L., Kneib T., Augustin T., Zeileis A. (2008). Conditional variable importance for random forests. *BMC Bioinformatics*.

[14] Göbl C. S., Bozkurt L., Tura A., Pacini G., Kautzky-Willer A., Mittlböck M. (2015). Application of penalized regression techniques in modelling insulin sensitivity by correlated metabolic parameters. *PLoS One*.

[15] Wagner R., Fritsche L., Heni M. (2016). A novel insulin sensitivity index particularly suitable to measure insulin sensitivity during gestation. *Acta Diabetologica*.

[16] Jones A. G., Hattersley A. T. (2013). The clinical utility of C-peptide measurement in the care of patients with diabetes. *Diabetic Medicine*.

