# Supplementary material for: Novel Indices of Glucose Homeostasis Derived from Principal Component Analysis: Application for Metabolic Assessment in Pregnancy
Source: J Diabetes Res. 2020 Mar 30;2020:4950584. doi: 10.1155/2020/4950584 (PMC7150715; doi:10.1155/2020/4950584)
Supplement: Supplementary Materials — Figure S1: box-whisker plots, representing comparisons of principal component scores between women who have normal glucose tolerance (NGT) and those who developed gestational diabetes mellitus (GDM): PCS1 (A); PCS2 (B); PCS3 (C). Table S1: loadings of each variable on the first three principal components (i.e., how strong original variables are related to each principal component), derived from our previous publication [1]. Table S2: correlation of the principal component scores (PCS) with glucometabolic parameters at 12+0 to 22+6 (V1) clearance rate; ISI: insulin sensitivity index; ISI-Matsuda: Matsuda index; OGIS: oral glucose insulin sensitivity index; PH1: first-phase insulin secretion; PH2: second-phase insulin secretion; Sec-early: early insulin response to glucose; Sec-late: late insulin response to glucose; Sec-tot: total insulin response to glucose; ISSI-2: disposition index; G-sens: β-cell glucose sensitivity; rate-sens: rate sensitivity; TIS: total insulin secretion from C-peptide. Table S3: correlation of the principal component scores (PCS) with glucometabolic parameters at 24+0 to 28+6 weeks of gestation (V2) clearance rate; ISI: insulin sensitivity index; ISI-Matsuda: Matsuda index; OGIS: oral glucose insulin sensitivity index; PH1: first-phase insulin secretion; PH2: second-phase insulin secretion; Sec-early: early insulin response to glucose; Sec-late: late insulin response to glucose; Sec-tot: total insulin response to glucose; ISSI-2: disposition index; G-sens: β-cell glucose sensitivity; rate-sens: rate sensitivity; TIS: total insulin secretion from C-peptide. Table S4: correlation of the principal component scores (PCS) with glucometabolic parameters 2 to 11 months after delivery (V3) clearance rate; ISI: insulin sensitivity index; ISI-Matsuda: Matsuda index; OGIS: oral glucose insulin sensitivity index; PH1: first-phase insulin secretion; PH2: second-phase insulin secretion; Sec-early: early insulin response to glucose; Sec-late: late insulin r [file 4950584.f1.docx]

**Figure S1:** Box-whisker plots, representing comparisons of principal component scores between women who remained normal glucose tolerant (NGT) and those who developed gestational diabetes mellitus (GDM): PCS1 (A); PCS2 (B); PCS3 (C)


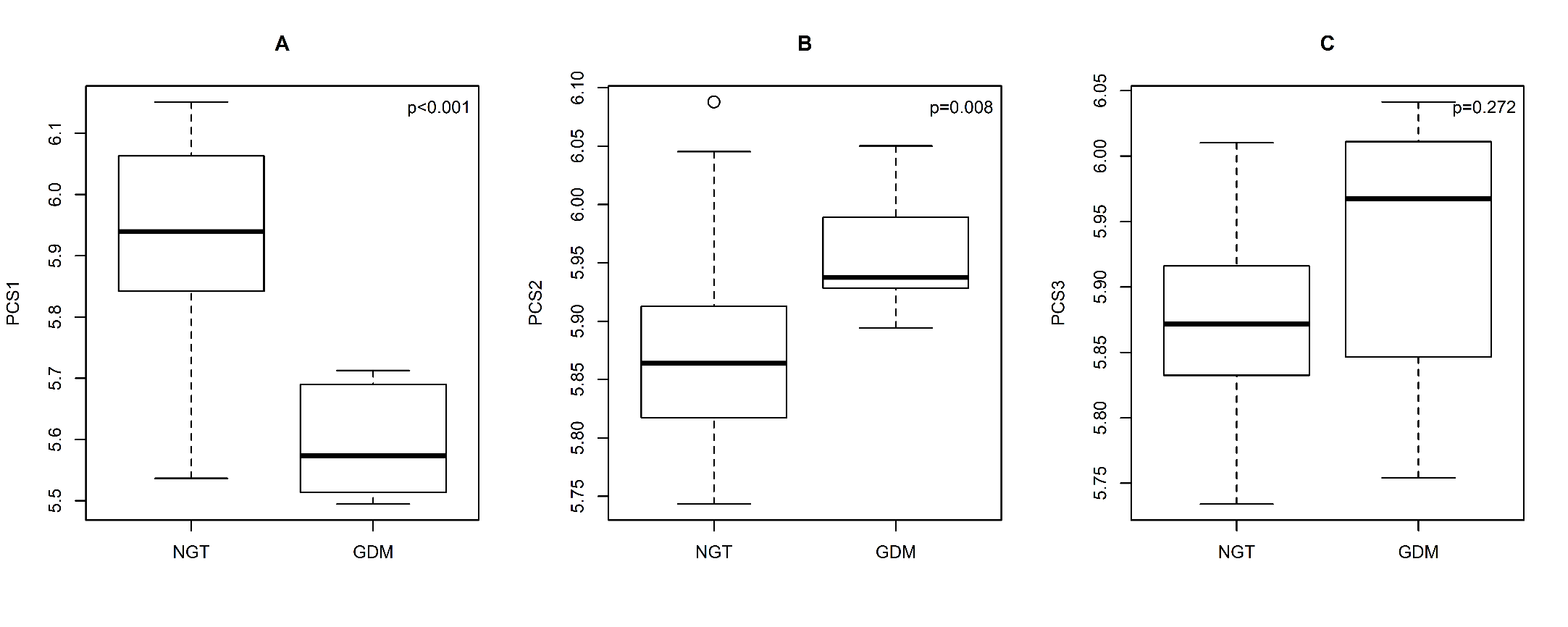


**Table S1:** Loadings of each variable on the first three principal components (i.e., how strong original variables are related to each principal component), derived from our previous publication (1)

|  | **PCS1** | **PCS2** | **PCS3** |
| --- | --- | --- | --- |
| G0 (mg/dl) | −0.1898 | 0.2192 | 0.4148 |
| G30 (mg/dl) | −0.2034 | 0.2673 | −0.0363 |
| G60 (mg/dl) | −0.2355 | 0.3647 | −0.1694 |
| G90 (mg/dl) | −0.2339 | 0.3830 | −0.0813 |
| G120 (mg/dl) | −0.1926 | 0.3540 | 0.0032 |
| I0 (µU/ml)* | −0.2588 | −0.1372 | 0.3487 |
| I30 (µU/ml)* | −0.1870 | −0.3843 | 0.0539 |
| I60 (µU/ml)* | −0.2816 | −0.1538 | −0.1749 |
| I90 (µU/ml)* | −0.2953 | −0.0123 | −0.2501 |
| I120 (µU/ml)* | −0.2875 | −0.0565 | −0.2029 |
| CP0 (ng/ml)** | −0.2755 | −0.0942 | 0.4221 |
| CP30 (ng/ml)** | −0.1969 | −0.3871 | 0.0949 |
| CP60 (ng/ml)** | −0.2592 | −0.2354 | −0.1340 |
| CP90 (ng/ml)** | −0.3045 | −0.0744 | −0.2637 |
| CP120 (ng/ml)** | −0.3084 | −0.0267 | −0.1973 |
| BMI (kg/m²)* | −0.2251 | 0.0443 | 0.4410 |
| Age (years) | −0.0618 | 0.2442 | 0.1566 |

BMI, body mass index. Glucose (G), Insulin (I) and C-peptide (CP) for fasting and 30, 60, 90 and 120 minutes after oral glucose load

* Log transformation (natural logarithm)

** Square root transformation

1. **Göbl CS**, **Bozkurt L**, **Mittlböck M**, **Leutner M**, **Yarragudi R**, **Tura A**, **Pacini G**, **Kautzky-Willer A**. To explain the variation of OGTT dynamics by biological mechanisms: a novel approach based on principal components analysis in women with history of GDM. *Am J Physiol Regul Integr Comp Physiol* 309: R13-21, 2015.

**Table S2:** Correlation of the principal component scores (PCS) with glucometabolic parameters at 12+0 to 22+6 (V1)

| **V1** | **PCS1** | | **PCS2** | | **PCS3** | |
| --- | --- | --- | --- | --- | --- | --- |
|  | rho | p-value | rho | p-value | rho | p-value |
| PCS1 | - | - | -0.14 | N.S. | 0.13 | N.S. |
| PCS2 | -0.14 | N.S. | - | - | 0.10 | N.S. |
| PCS3 | 0.13 | N.S. | 0.10 | N.S. | - | - |
| G-mean | **-0.83** | **<0.001** | **0.57** | **<0.001** | -0.10 | N.S. |
| I-mean | **-0.88** | **<0.001** | -0.06 | N.S. | -0.22 | N.S. |
| CP-mean | **-0.91** | **<0.001** | -0.07 | N.S. | **-0.25** | **0.045** |
| HOMA-IR | **-0.73** | **<0.001** | -0.06 | N.S. | **0.35** | **0.004** |
| QUICKI | **0.73** | **<0.001** | -0.06 | N.S. | **-0.35** | **0.004** |
| MCR | **0.87** | **<0.001** | **-0.28** | **0.022** | -0.03 | N.S. |
| ISI | **0.88** | **<0.001** | **-0.29** | **0.019** | -0.02 | N.S. |
| ISI-Matsuda | **0.93** | **<0.001** | -0.06 | N.S. | -0.01 | N.S. |
| OGIS | **0.78** | **<0.001** | -0.19 | N.S. | 0.02 | N.S. |
| PH1 | **-0.65** | **<0.001** | **-0.37** | **0.002** | 0.04 | N.S. |
| PH2 | **-0.69** | **<0.001** | **-0.32** | **0.008** | 0.06 | N.S. |
| Sec-early | **-0.40** | **<0.001** | **-0.48** | **<0.001** | -0.01 | N.S. |
| Sec-late | **-0.78** | **<0.001** | -0.16 | N.S. | **-0.33** | **0.006** |
| Sec-total | **-0.79** | **<0.001** | **-0.26** | **0.032** | -0.21 | N.S. |
| ISSI-2 | **0.39** | **0.001** | **-0.52** | **<0.001** | -0.48 | **<0.001** |
| G-sens | 0.01 | N.S. | **-0.67** | **<0.001** | -0.03 | N.S. |
| rate-sens | -0.03 | N.S. | **-0.29** | **0.016** | -0.18 | N.S. |
| TIS | **-0.88** | **<0.001** | -0.10 | N.S. | **-0.35** | **0.004** |

Abbreviations are rho, Spearman’s rank correlation; PCS, principal component scores; G, glucose; I, Insulin; CP, C-peptide; HOMA-IR, homeostatic model assessment of insulin resistance; QUICKI, quantitative insulin sensitivity check index; MCR, metabolic clearance rate; ISI, insulin sensitivity index; ISI-Matsuda, Matsuda index; OGIS, oral glucose insulin sensitivity index; PH1, first phase insulin secretion; PH2, second phase insulin secretion; Sec-early, early insulin response to glucose, Sec-late, late insulin response to glucose; Sec-tot, total insulin response to glucose; ISSI-2, disposition index; G-sens, β-cell glucose sensitivity; rate-sens, rate sensitivity; TIS, total insulin secretion from C-peptide

**Table S3:** Correlation of the principal component scores (PCS) with glucometabolic parameters at 24+0 to 28+6 weeks of gestation (V2)

| **V2** | **PCS1** | | **PCS2** | | **PCS3** | |
| --- | --- | --- | --- | --- | --- | --- |
|  | rho | p-value | rho | p-value | rho | p-value |
| PCS1 | - | - | 0.03 | N.S. | 0.10 | N.S. |
| PCS2 | 0.03 | N.S. | - | - | 0.00 | N.S. |
| PCS3 | 0.10 | N.S. | 0.00 | N.S. | - | - |
| G-mean | **-0.71** | **<0.001** | **0.52** | **<0.001** | -0.09 | N.S. |
| I-mean | **-0.85** | **<0.001** | -0.26 | N.S. | -0.19 | N.S. |
| CP-mean | **-0.87** | **<0.001** | -0.17 | N.S. | -0.11 | N.S. |
| HOMA-IR | **-0.50** | **<0.001** | -0.24 | N.S. | **0.28** | **0.035** |
| QUICKI | **0.50** | **<0.001** | 0.24 | N.S. | **-0.28** | **0.035** |
| MCR | **0.75** | **<0.001** | -0.12 | N.S. | -0.07 | N.S. |
| ISI | **0.75** | **<0.001** | -0.13 | N.S. | -0.06 | N.S. |
| ISI-Matsuda | **0.81** | **<0.001** | 0.23 | N.S. | -0.05 | N.S. |
| OGIS | **0.80** | **<0.001** | -0.03 | N.S. | 0.22 | N.S. |
| PH1 | **-0.51** | **<0.001** | **-0.56** | **<0.001** | 0.14 | N.S. |
| PH2 | **-0.54** | **<0.001** | **-0.53** | **<0.001** | 0.14 | N.S. |
| Sec-early | **-0.33** | **0.011** | **-0.45** | **<0.001** | 0.11 | N.S. |
| Sec-late | **-0.65** | **<0.001** | **-0.37** | **0.004** | **-0.33** | **0.012** |
| Sec-total | **-0.70** | **<0.001** | **-0.48** | **<0.001** | -0.21 | N.S. |
| ISSI-2 | 0.12 | N.S. | **-0.44** | **<0.001** | **-0.41** | **0.002** |
| G-sens | -0.23 | N.S. | **-0.57** | **<0.001** | 0.07 | N.S. |
| rate-sens | -0.05 | N.S. | **-0.41** | **0.001** | -0.12 | N.S. |
| TIS | **-0.86** | **<0.001** | -0.17 | N.S. | -0.19 | N.S. |

Abbreviations are rho, Spearman’s rank correlation; PCS, principal component scores; G, glucose; I, Insulin; CP, C-peptide; HOMA-IR, homeostatic model assessment of insulin resistance; QUICKI, quantitative insulin sensitivity check index; MCR, metabolic clearance rate; ISI, insulin sensitivity index; ISI-Matsuda, Matsuda index; OGIS, oral glucose insulin sensitivity index; PH1, first phase insulin secretion; PH2, second phase insulin secretion; Sec-early, early insulin response to glucose, Sec-late, late insulin response to glucose; Sec-tot, total insulin response to glucose; ISSI-2, disposition index; G-sens, β-cell glucose sensitivity; rate-sens, rate sensitivity; TIS, total insulin secretion from C-peptide

**Table S4:** Correlation of the principal component scores (PCS) with glucometabolic parameters 2 to 11 months after delivery (V3)

| **V3** | **PCS1** | | **PCS2** | | **PCS3** | |
| --- | --- | --- | --- | --- | --- | --- |
|  | rho | p-value | rho | p-value | rho | p-value |
| PCS1 | - | - | -0.08 | N.S. | -0.09 | N.S. |
| PCS2 | -0.08 | N.S. | - | - | 0.18 | N.S. |
| PCS3 | -0.09 | N.S. | 0.18 | N.S. | - | - |
| G-mean | **-0.70** | **<0.001** | **0.62** | **0.002** | 0.25 | N.S. |
| I-mean | **-0.64** | **0.001** | **-0.43** | **0.040** | -0.30 | N.S. |
| CP-mean | **-0.81** | **<0.001** | -0.12 | N.S. | 0.05 | N.S. |
| HOMA-IR | **-0.56** | **0.006** | -0.04 | N.S. | **0.51** | **0.012** |
| QUICKI | **0.56** | **0.006** | 0.04 | N.S. | **-0.51** | **0.012** |
| MCR | **0.63** | **0.001** | -0.21 | N.S. | -0.13 | N.S. |
| ISI | **0.65** | **<0.001** | -0.26 | N.S. | -0.12 | N.S. |
| ISI-Matsuda | **0.65** | **<0.001** | 0.25 | N.S. | -0.24 | N.S. |
| OGIS | **0.54** | **0.009** | -0.06 | N.S. | **-0.46** | **0.031** |
| PH1 | -0.05 | N.S. | **-0.60** | **0.002** | 0.08 | N.S. |
| PH2 | -0.14 | N.S. | **-0.59** | **0.003** | 0.10 | N.S. |
| Sec-early | -0.24 | N.S. | -0.30 | N.S. | 0.05 | N.S. |
| Sec-late | **-0.49** | **0.017** | **-0.56** | **0.006** | -0.32 | N.S. |
| Sec-total | **-0.45** | **0.033** | **-0.58** | **0.004** | -0.20 | N.S. |
| ISSI-2 | 0.40 | N.S. | -0.35 | N.S. | **-0.69** | **<0.001** |
| G-sens | -0.37 | N.S. | -0.06 | N.S. | **-0.44** | **0.034** |
| rate-sens | -0.20 | N.S. | 0.05 | N.S. | 0.30 | N.S. |
| TIS | **-0.81** | **<0.001** | -0.17 | N.S. | -0.06 | N.S. |

Abbreviations are rho, Spearman’s rank correlation; PCS, principal component scores; G, glucose; I, Insulin; CP, C-peptide; HOMA-IR, homeostatic model assessment of insulin resistance; QUICKI, quantitative insulin sensitivity check index; MCR, metabolic clearance rate; ISI, insulin sensitivity index; ISI-Matsuda, Matsuda index; OGIS, oral glucose insulin sensitivity index; PH1, first phase insulin secretion; PH2, second phase insulin secretion; Sec-early, early insulin response to glucose, Sec-late, late insulin response to glucose; Sec-tot, total insulin response to glucose; ISSI-2, disposition index; G-sens, β-cell glucose sensitivity; rate-sens, rate sensitivity; TIS, total insulin secretion from C-peptide

**Table S5:** Association with development of GDM and variable importance index provided by random forest analysis

|  | OR | 95%CI | p-value | VIMP |
| --- | --- | --- | --- | --- |
| PCS1 | 0.82 | 0.68-9.10 | <0.001 | 1.54×10^-2^ |
| PCS2 | 1.18 | 1.05-1.37 | 0.004 | 6.67×10^-7^ |
| PCS3 | 1.14 | 1.00-1.32 | 0.046 | -7.50×10^-7^ |
| age (years) | 1.47 | 1.17-2.06 | <0.001 | -1.13×10^-5^ |
| BMI (kg/m²) | 1.38 | 1.16-1.74 | <0.001 | 3.40×10^-3^ |
| family history with diabetes | 2.00 | 0.34-11.7 | 0.424 | 0.00 |
| G0 (mg/dl) | 1.35 | 1.15-1.71 | <0.001 | 3.06×10^-3^ |
| G-mean (mg/dl) | 1.18 | 1.08-1.41 | <0.001 | 8.17×10^-3^ |
| OGIS (ml min-1m-2) | 0.98 | 0.96-0.91 | <0.001 | 1.67×10^-6^ |
| TIS (nmol m-2) | 1.09 | 1.03-1.16 | 0.002 | -3.58×10^-5^ |

Odds ratios (OR) and 95% Confidence Intervals (95%CI); VIMP, variable
importance measure from random forest analysis; PCS, principal component scores; BMI, body mass index; G0, fasting glucose; G-mean, mean glucose during the OGTT; OGIS, oral glucose insulin sensitivity index; TIS, total insulin sensitivity index from C-peptide; all data was assessed at the first visit (12+0 to 22+6)
